# Nilotinib in *KIT*-driven advanced melanoma: Results from the phase II single-arm NICAM trial

**DOI:** 10.1016/j.xcrm.2024.101435

**Published:** 2024-02-27

**Authors:** James Larkin, Richard Marais, Nuria Porta, David Gonzalez de Castro, Lisa Parsons, Christina Messiou, Gordon Stamp, Lisa Thompson, Kim Edmonds, Sarah Sarker, Jane Banerji, Paul Lorigan, Thomas R. Jeffry Evans, Pippa Corrie, Ernest Marshall, Mark R. Middleton, Paul Nathan, Steve Nicholson, Christian Ottensmeier, Ruth Plummer, Judith Bliss, Sara Valpione, Samra Turajlic

**Affiliations:** 1 Skin and Renal Units, The Royal Marsden Hospital NHS Foundation Trust, London, UK; 2 Melanoma and Kidney Cancer Team, The Institute of Cancer Research, London, UK; 3 Cancer Research UK Manchester Institute, The University of Manchester, Manchester, UK; 4 Clinical Trials and Statistics Unit, The Institute of Cancer Research, London, UK; 5 Molecular Diagnostics, The Institute of Cancer Research and Royal Marsden NHS Foundation Trust, London, UK; 6 University of Edinburgh, Edinburgh, UK; 7 PDD - Thermo Fisher Scientific, Bend, Oregon, USA; 8 Department of Radiology, The Royal Marsden Hospital NHS Foundation Trust, London, UK; 9 Department of Histopathology, The Royal Marsden Hospital NHS Foundation Trust, London, UK; 10 Centre for Molecular Pathology, The Royal Marsden Hospital NHS Foundation Trust, London, UK; 11 Division of Cancer Sciences, Unviersity of Manchester, Manchester, UK; 12 The Christie NHS Foundation Trust, Manchester, UK; 13 Institute of Cancer Sciences, University of Glasgow, Glasgow, UK; 14 Cambridge University Hospitals NHS Foundation Trust, Cambridge, UK; 15 The Clatterbridge Cancer Centre NHS Foundation Trust, Liverpool, UK; 16 Department of Oncology, University of Oxford, Oxford, UK; 17 Mount Vernon Cancer Centre, East & North Herts NHS Trust, Northwood, UK; 18 University Hospitals of Leicester NHS Foundation Trust, Leicester, UK; 19 University Hospitals Southampton NHS Foundation Trust, Southampton, UK; 20 Newcastle University and Newcastle Upon Tyne Hospitals NHS Foundation Trust, Newcastle, UK; 21 Cancer Dynamics Laboratory, The Francis Crick Institute, London, UK

**Keywords:** *KIT* mutation, tyrosine kinase inhibitor, melanoma, mucosal, acral, liquid biopsy

## Abstract

Mucosal (MM) and acral melanomas (AM) are rare melanoma subtypes of unmet clinical need; 15%–20% harbor *KIT* mutations potentially targeted by small-molecule inhibitors, but none yet approved in melanoma. This multicenter, single-arm Phase II trial (NICAM) investigates nilotinib safety and activity in *KIT* mutated metastatic MM and AM. *KIT* mutations are identified in 39/219 screened patients (18%); of 29/39 treated, 26 are evaluable for primary analysis. Six patients were alive and progression free at 6 months (local radiology review, 25%); 5/26 (19%) had objective response at 12 weeks; median OS was 7.7 months; ddPCR assay correctly identifies *KIT* alterations in circulating tumor DNA (ctDNA) in 16/17 patients. Nilotinib is active in *KIT*-mutant AM and MM, comparable to other KIT inhibitors, with demonstrable activity in nonhotspot *KIT* mutations, supporting broadening of *KIT* evaluation in AM and MM. Our results endorse further investigations of nilotinib for the treatment of *KIT*-mutated melanoma. This clinical trial was registered with ISRCTN (ISRCTN39058880) and EudraCT (2009-012945-49).

## Highlights

• Whole-gene *KIT* sequencing identified mutations in 18% of advanced acral and mucosal melanomas
• Nilotinib induced objective responses at 12 weeks in 19% of patients with *KIT*-mutated melanoma
• Droplet digital PCR assays are a feasible liquid biopsy for *KIT* mutations

## Graphical abstract

**Figure:**
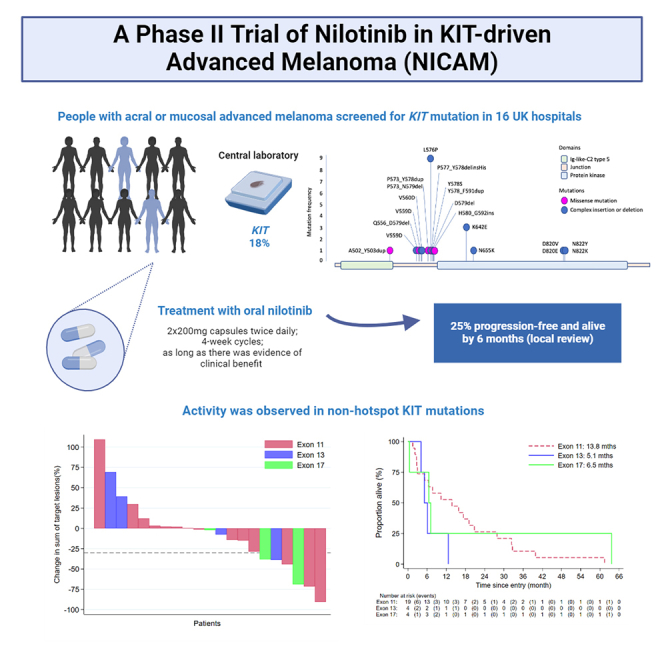

## Teaser

Larkin et al. found that 6/26 patients with a rare melanoma had not progressed after ≥6 months of treatment with nilotinib. These rare melanomas have a mutation in a gene called *KIT*, and this gene abnormality was also found in the DNA that circulates in plasma.

## Introduction

Acral melanomas (AM) and mucosal melanomas (MM) are rare subtypes of melanomas (comprising ∼5%), and arise from nonglabrous skin, including mucosa (MM), soles, palms, and the nail bed (AM).^1^^,^^2^ MM and AM are clinically and genetically distinct from the common cutaneous melanomas. MM exhibit aggressive clinical behavior, commonly recur after surgical removal, resulting in 5-year survival rates of just 14%, compared to 90% 5-year survival of patients with cutaneous melanomas.^3^^,^^4^ AM have inferior outcomes compared to UV-associated cutaneous melanomas,^2^^,^^5^^,^^6^^,^^7^ due to frequently delayed diagnosis and inherently more aggressive disease course.^8^

UV-driven mutagenesis is limited in AM and only found in a small proportion of MM from sun-exposed mucosa, including the conjunctiva and lips.^3^^,^^9^^,^^10^^,^^11^^,^^12^^,^^13^^,^^14^^,^^15^^,^^16^ MM and AM have low tumor mutational burden, and instead are characterized by higher levels of chromosomal complexity.^16^ *BRAF* mutations, present in ∼40%–50% of common cutaneous melanomas,^17^ are detected in ∼20% AM^18^^,^^19^^,^^20^ and are largely absent in MM^21^^,^^22^; thus, only a minority of these patients are suitable for treatment with BRAF&MEK targeting agents. Immune checkpoint blockade (ICB) has transformed the outcomes of patients with cutaneous metastatic melanoma with 5-year survival rates of ∼50%^23^; however, the proportion of patients with AM and MM who benefit from ICB is significantly lower by comparison—the programmed death-1 (PD-1) blockade response rate of 15%–40% versus 40%–50% and overall survival of 11.5 versus 25.8 months.^12^^,^^24^^,^^25^^,^^26^^,^^27^ Thus, AM and MM have relatively limited treatment options, further aggravated by the disease rarity, frequent exclusion from Phase III clinical trials, and lack of evidence base for clinical decision making.

The aim of this study was to evaluate the efficacy of nilotinib in advanced *KIT*-mutated melanoma, to explore the particularities of *KIT* mutation and copy number amplification and benefit from treatment, and to assess the value of droplet digital PCR (ddPCR) for the liquid biopsy of melanomas with uncommon *KIT* mutations and complex aberrations.

## Results

### Patients

**Figure 1:**
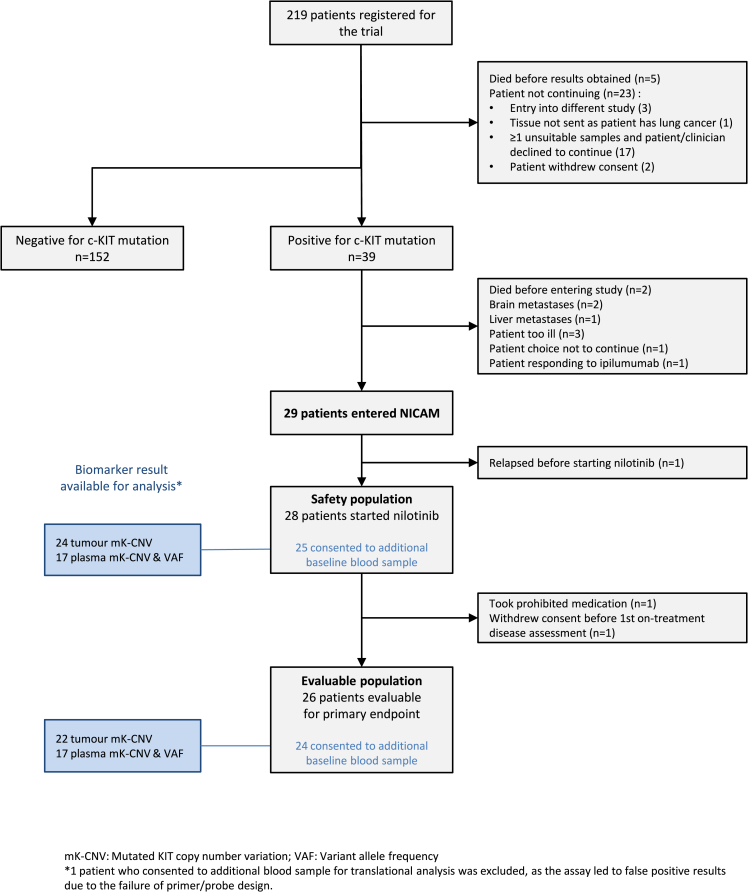
Patient flowchart in the NICAM trial

**Figure 2:**
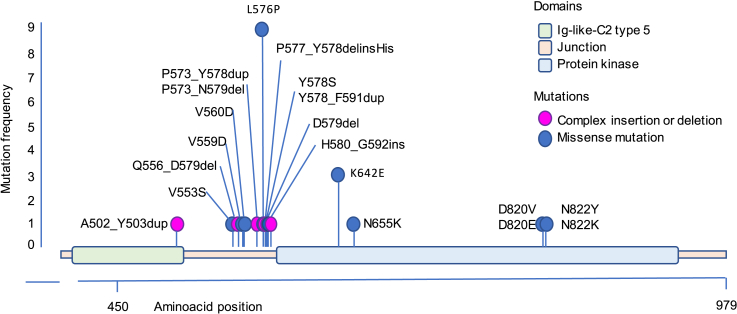
c-*KIT* molecular characterization in the NICAM trial The chart shows the individual c-*KIT* mutation characterization in the 29 trial patients who were enrolled in the molecular profiling. The gene fragment affected by mutations spanned from exons 9 to 17, comprising the immunoglobulin-like-C2 type 5 domain (green), a junction domain (pink), and the protein kinase domain (light blue). Each lollipop anchor corresponds to individual mutation sites (complex mutations are in purple and missense single-nucleotide mutations are in blue), and the height of the lollipop is indicative of the mutation frequency in the trial population.

Between December 15, 2009 and August 4, 2014, 219 patients with the diagnosis of advanced AM or MM meeting eligibility criteria were screened for the presence of *KIT* mutations. *KIT* mutations were detected in 39 (18%) patients, 29 (13%) of which were considered eligible to enter the treatment part of the trial (Figure 1). One of the 10 ineligible patients was excluded due to the finding of the exon 17 *KIT* mutation that likely conferred resistance to nilotinib based on prior reports.^28^ Baseline characteristics of enrolled patients are shown in Table 1 (see Table S1 for baseline features of all screened patients). Six patients presented with AM (20.7%), and 23 with MM (79.3%). *KIT* mutations were found in exon 11 (n = 20, 69%), exon 13 (n = 4, 14%), exon 17 (n = 4.14%), and exon 9 (n = 1, 3%). A total of 21 (72%) mutations were single-nucleotide variants, whereas 8 (28%) were insertions or deletions (indels). The most common mutation was L576, which we observed in 9 patients (31%) (Figure 2; Table S2).

**Table 1:** Baseline characteristics of patients entered into the NICAM trial (N = 29)

	N	%
**Patient demographics**		

Sex		
Female	20	69
Male	9	31
Age at registration/entry (yr), mean (SD)	67.1 (9.1)	
Ethnicity		
White	22	75.9
Asian	2	6.9
Other	4	13.8
Unknown	1	3.4
Skin type (Fitzpatrick classification)		
I	3	10.3
II	1	3.4
III	17	58.6
IV	3	10.3
V	1	3.4
VI	2	6.9
Unknown	2	6.9

**Disease at presentation and past treatments**		

Melanoma subtype		
Acral	6	20.7
Location		
Hand	1	3.4
Foot	5	17.3
Stage at presentation		
Localized	3	10.3
Regional lymph node metastasis	2	6.9
Unknown	1	3.4
Mucosal	23	79.3
Location		
Head and neck	5	17.2
Upper gastrointestinal tract	2	6.9
Anorectal	5	17.2
Urogenital	11	37.9
Other^a^	1	3.4
Stage at presentation		
Localized I	6	20.7
Localized II	7	24.1
Localized III	1	3.4
Unknown	9	31.0
Prior treatments		
Radiotherapy	9	31
Systemic treatment (palliative)^b^^,^^c^	4	13.8

**Disease at trial entry**		

Time from diagnosis (yr) to trial entry, median (Q1–Q3)	1.3 (0.7–3.3)	
ECOG performance status		
0	16	55.2
1	12	41.4
2	1	3.4
Location of disease^c^		
Local	5	17.2
Lymph nodes	20	69.0
Liver	11	37.9
Lung	21	72.4
Brain	0	0
Other	8	27.6
Disease burden at trial entry (sum of target lesions in cm as per RECIST 1.1), median (Q1–Q3)	7.2 (4.8–10.5)	
LDH at trial entry (U/L), median (Q1–Q3), N = 25	259 (199–358)	

ECOG, Eastern Cooperative Oncology Group; LDH, lactate dehydrogenase.

a One patient specified two primary sites (urogenital and other–unknown).

b Includes immunotherapy (n = 3): interferon and interleukin-2 (n = 1), ipilimumab (n = 1), other (n = 1); chemotherapy (n = 3).

c More than one option per patient could be specified.

**Figure 3:**
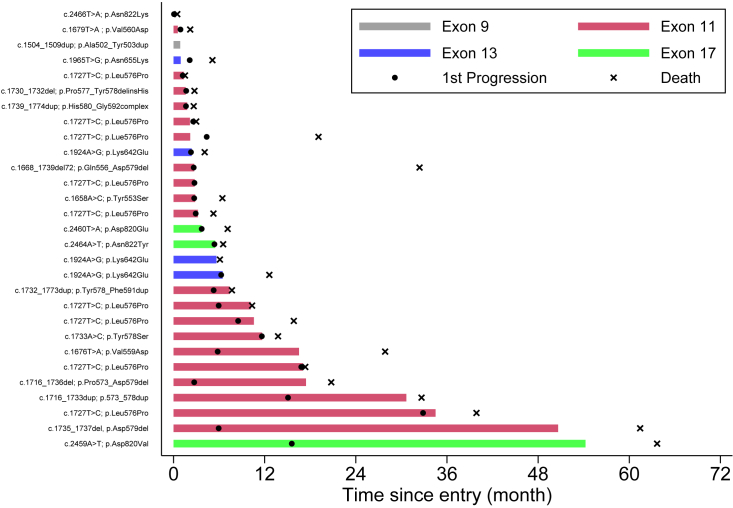
Time on treatment for all entered NICAM patients, by cKIT mutation exon Bar length indicate months on treatment; objective disease progression and death are indicated in the figure. Patients were allowed to continue treatment as long as clinically indicated by the treating physician.

Among the patients who received at least one dose of nilotinib (n = 28), the median time on treatment was 3.7 months (first to third quartiles [Q1–Q3], 2.2–11.7 months) (Figure 3). One patient remained on treatment for more than 50 months. Overall, 22 patients (79%) had at least one dose reduction, delay, or missed treatment (Figure S1); of these, 8 patients (29%) had at least one nilotinib dose reduction (4/8 due to abnormal liver function, 2/8 due to other toxicities, 2/8 due to omitting doses in error). At data cutoff, the median follow-up for patients on trial was 7.1 months (Q1–Q3, 3.0–19.1 months).

Overall, 26 patients were evaluable for the primary endpoint. Three unevaluable participants (all MM) included one who discontinued due to toxicity before the first scan, but was deemed unevaluable because the individual was taking a prohibited concomitant medication; one patient who withdrew consent for all trial procedures after 1.4 months of treatment; and one who progressed before receiving any trial treatment.

### Safety

All of the patients who received at least one dose of nilotinib (n = 28) were assessed for safety. National Cancer Institute-Common Terminology Criteria (NCI-CTC) grade 3 adverse events (AEs) or higher were reported in 18 patients (64%) while on treatment (Table 2). The most frequent AEs of any grade were fatigue (N = 21, 75%), nausea (n = 17, 61%) and constipation (n = 14, 50%). A total of 16 serious AEs in 10 patients were reported, of which only two events in one patient were deemed to be related to study drug (SAR). This patient experienced both SAR within 2 months of commencing treatment (raised alanine aminotransferase grade 4, aspartate aminotransferase grade 3, and bilirubin grade 2) and permanently discontinued nilotinib. We note that this patient had been taking concomitant prohibited herbal medication, which may have contributed to the liver dysfunction. A further patient experienced a treatment-related toxicity (deranged liver function), leading to 50% dose reduction and then treatment discontinuation. There were no treatment-related deaths.

**Table 2:** Treatment-emergent adverse events in the NICAM trial (N = 28, safety population)

	Grade 1+		Grade 3+	
	N	%	N	%
Fatigue	21	75.0	3	10.7
Nausea	17	60.7	2	7.1
Constipation	14	50.0	1	3.6
Rash	12	42.9	0	0.0
Anorexia	12	42.9	0	0.0
Anemia	10	35.7	1	3.6
Vomiting	8	28.6	2	7.1
Alopecia	8	28.6	0	0.0
Abdominal pain	7	25.0	3	10.7
Diarrhea	6	21.4	1	3.6
Arthralgia	6	21.4	1	3.6
Bone pain	6	21.4	0	0.0
Peripheral edema	6	21.4	0	0.0
Pruritus	6	21.4	0	0.0
Headache	4	14.3	1	3.6

The above toxicities were prespecified in the Case Report Form (CRF) at each cycle; additional toxicities graded 3+ not prespecified in the CRF were observed in 12 patients: alanine aminotransferase increased (1, 4%), aspartate aminotransferase increased (1.4%), back pain (1, 4%), blood LDH increased (1, 4%), breast cancer female (1, 4%), cellulitis (2, 8%), chest pain (1, 4%), convulsion (1, 4%), deep vein thrombosis (1, 4%), dehydration (1, 4%), dyspnea (1, 4%), embolism (1, 4%), hypertension (1, 4%), lower respiratory tract infection (2, 8%), muscular weakness (1, 4%), esophageal pain (1, 4%), pain (1, 4%), pleural effusion (1, 4%), pneumonia (1, 4%), urogenital hemorrhage (1, 4%).

### Antitumor activity

Of the first 24 evaluable patients as prespecified in the two-stage design, six patients were progression free at 6 months as reported locally (25% 90% confidence interval [CI] 12–44, p = 0.11), thus not fulfilling the prespecified success criteria. However, central review of the primary endpoint indicated that there were seven patients who were progression free at 6 months (29%, 90% CI 15–47, p = 0.05). Accounting for the two-stage design, the local and central estimate of 6-month progression-free survival (PFS) were, respectively, 30% and 33%. Over all 26 evaluable patients, the estimates for 6-month PFS rate accounting for the two-stage design were 29% (90% CI: 11–44, p = 0.14) as per local review and 31% (90% CI 14–45) as per central review. Of note, all of the acral-subtype patients progressed by 6 months.

Objective response evaluation criteria in solid tumors (RECIST) 1.1 objective response (OR) at 12 weeks was 5/26 patients (19% [95% CI 7–39]) based on local reporting. Median PFS was 3.7 months (95% CI 2.7–5.9), and PFS at 6 months as estimated by Kaplan-Meier (Figure S2) was 23% (95% CI 9–40). Median overall survival (OS) was 7.7 months (95% CI 5.3–17.3); OS at 12 months was 44% (95% CI 25–62) (Figure S2). Disease burden at baseline (measured by the sum of target lesion diameters, in cm) was not statistically associated with PFS (hazard ratio [HR] = 1.04 [95% CI 0.96–1.11], p = 0.34) but it was associated with worse OS (HR = 1.08 [95% CI 1.00–1.16], p = 0.043). Acral tumors had worse median PFS (2.3 months) and OS (5.1 months) than mucosal tumors (PFS 5.4 months, OS 7.7 months), although differences were not significant.

The presence of indolent disease at baseline could be centrally reviewed in 19 patients in whom prebaseline scans were available. Of these, 4/19 (21%) presented indolent disease at baseline (see STAR Methods), but only one patient with indolent disease was alive and progression free at 6 months. It does not seem, therefore, that indolent disease is driving the observed response to nilotinib.

### Association of KIT mutation and gene amplification with antitumor activity

**Figure 4:**
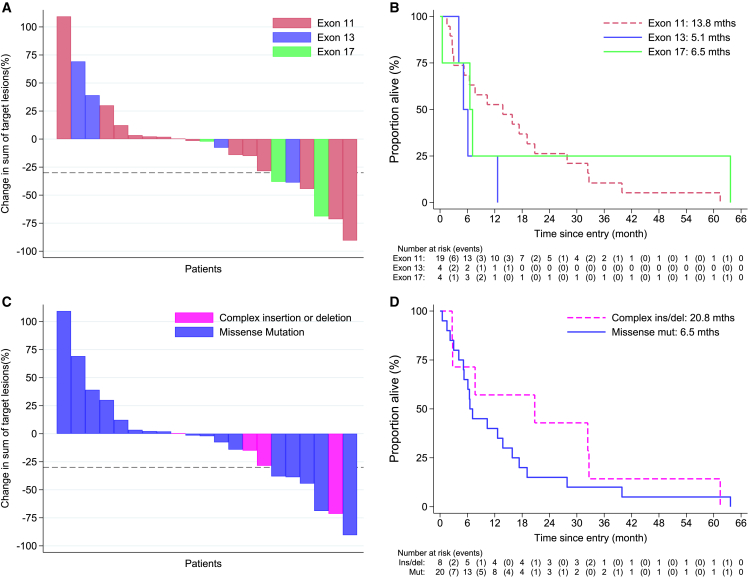
Association of mutation with outcome data (A) Percentage change from baseline at 12 weeks in sum of target lesions as per RECIST 1.1 by exon where *KIT* mutation was detected. (B) OS by exon where *KIT* mutation was detected. (C) Percentage change from baseline at 12 weeks in sum of target lesions as per RECIST 1.1 by type of *KIT* mutation. (D) OS by type of *KIT* mutation. For the waterfall plots (A) and (C), only evaluable patients with data at the 12-week scan since start of nilotinib are included.

Central assessment of antitumor activity was used for the following association analyses. No significant differences according to the exon in which the *KIT* mutation were observed in OR at 12 weeks (exon 11: 3/19 [16%]; exon: 13 1/4 [25%]; exon 17: 2/3 [67%], p = 0.15) or median PFS (exon 11: 2.9 months; exon 13: 2.3 months; exon 17: 5.4 months; p = 0.75) (Figure 4A). Median OS was 13.8 months for patients with mutations in exon 11, 5.1 months in exon 13, and 6.5 months in exon 17, although the differences were not significant (Figure 4B, p = 0.26). Note that three out of four mutations found in exon 13 corresponded to acral tumors (Table S2). We observed an outlier patient with D820V *KIT* mutation (exon 17) who remained on treatment for 54 months. In terms of mutational class, the OR rate at 12 weeks was 14.3% (1/7) in patients with complex indels and 26.3% (5/19) in patients with single-nucleotide variants (Figure 4C), with no significant difference found in median PFS (2.7 months vs. 5.4, p = 0.38) nor OS (20.8 vs. 6.5, p = 0.34; Figure 4D).

Mutated *KIT* copy number (mK-CN; measured on baseline biopsy sample) tumor values (see STAR Methods), reflecting copy-number status of the *KIT* gene, could be inferred in 22 evaluable patients in baseline tumor samples. Median mK-CN was 3.5 (Q1–Q3, 1.3–7.1; Figure S3A), consistent with the presence of high-level *KIT* amplification in a subset of patients (see type of mutation by mK-CN amplification in Table S3). We did not find a significant correlation between tumor mK-CN and overall disease burden at baseline (Figure S3B).

There was no significant difference in the distribution of mK-CN between patients with OR at 12 weeks compared to nonresponders (Figure S3C, p = 0.56). mK-CN (considered continuous variable, centered to its mean and scaled by its SD) was not significantly associated with PFS (HR = 0.98 [95% CI 0.63–1.53], p = 0.93) nor OS (HR = 1.08 [95% CI 0.68–1.73], p = 0.73). Median PFS was 3.7 months in patients with mK-CN at or above the median (amplified) compared to 5.3 months in patients with mK-CN below the median (nonamplified, p = 0.73). Median OS was 7.1 and 7.7 months, respectively (Figure S3D, p = 0.64). Best tumor shrinkage at 12 weeks by type of mutation and amplification is presented in Figure S4.

To explore the intratumor heterogeneity of *KIT* amplification, we performed fluorescence *in situ* hybridization (FISH) in six evaluable samples (Table S4), observing some degree of heterogeneity in at least one case (Figure S5), with mean *KIT* copies = 5.9. Table S4 also refers to whole-genome and exome sequencing performed for two and four patients in the trial.

### Mutation analysis in plasma

Finally, we explored the feasibility of ddPCR testing to identify *KIT* alterations in plasma. For this purpose, baseline blood samples were available for 18 evaluable patients. The design of specific primer/probes for mutation analysis in circulating tumor DNA (ctDNA) and matched formalin-fixed paraffin embedded (FFPE) tumor was successful for all but one patient, in whom ddPCR could not satisfactorily differentiate the wild type and the complex indel mutated sequence. The concordance of mutations detected in ctDNA and FFPE tumor was 100%.

*KIT* VAF_adj_, which is the frequency of the variant allele in plasma, adjusted for mK-CN, could be inferred in all 17 blood samples. We did not find a significant correlation between VAF_adj_ and overall disease burden at baseline (Figure S6A). There was no significant difference in baseline plasma VAF_adj_ between responders and nonresponders (Figure S6B). Baseline plasma VAF_adj_ (as a continuous variable, centered to its mean and scaled to its SD) was not significantly associated with PFS (HR = 0.70 [95% CI 0.37–1.31], p = 0.27) nor OS (HR = 0.94 [95% CI 0.58–1.53], p = 0.82).

## Discussion

Rare cancers pose a unique challenge for the clinical development of new therapies because the scarcity of appropriate patient populations makes it difficult to perform sufficiently powered studies to gain evidence.^29^^,^^30^ The advent of molecular stratification and personalized medicine such as the current approaches for BRAF mutant cutaneous melanoma and *KIT*-mutant gastrointestinal tumors offer hope to these patients. However, additional challenges exist in the setting of a rare cancer with infrequent targetable alterations.^31^ This is evident in our study, in which 219 patients were screened, with only 29 entering the trial.

Mutations in the stem cell factor receptor gene *KIT* are reported in ∼5%–20% of AM and MM^11^^,^^12^^,^^15^^,^^16^^,^^32^^,^^33^ and is the sole currently targetable molecular alteration in these patients. Mutant KIT targeting has been trialled with varied success, with response rates ranging from 0% to 26% (Table S5).^34^^,^^35^^,^^36^^,^^37^^,^^38^^,^^39^^,^^40^^,^^41^^,^^42^^,^^43^^,^^44^ Critically, the impact of the *KIT* mutation type, especially outside exon 11, and the additional presence of *KIT* amplification, on the treatment response has not been investigated prospectively. Moreover, the utility of ctDNA analysis, which is established for the more common melanoma genotypes^45^^,^^46^ is explored only to a limited degree in *KIT*-mutated melanomas.^47^

Our data show that nilotinib has activity in the setting of *KIT* mutant melanoma, comparable to other KIT inhibitors with toxicity profiles consistent with previous reports.^42^ Despite the time lapse since the study conception and the advancements in the analytical technologies that have become available, there have been no breakthrough advances in terms of targeted therapy for AM and MM, no dedicated randomized Phase III trials, and KIT inhibitors remain unlicensed in most countries. Our results will, therefore, add to the body of evidence to plan future trials in these cancers of unmet need.

We also show that ddPCR in the plasma can accurately pinpoint^40^ the tumor mutational profile. Additionally, we showed that tumor-informed ddPCR is a feasible and reliable tool for evaluating *KIT* aberrations, including complex indels; hence, we propose that it could be implemented in future personalized oncology strategies, such as disease-response monitoring and minimal residual disease assessment in the adjuvant setting of AM and MM. The findings regarding the prognostic value of plasma mk-CN require validation but nonetheless warrant further investigations. Similar to our findings, the concomitant *KRAS* mutation and amplification has a predictive effect for greater benefit from treatment in *KRAS*-mutated lung cancers,^48^ and high allele fraction for *BRAF* mutation, which is an adverse prognostic factor in colorectal cancers, is associated with a higher benefit from triplet therapy with EGFR-BRAF-MEK inhibitors (OS HR = 0.17) compared to the cancers with low *BRAF* mutation allele frequency cancers (OS HR = 0.90).^49^ Concomitant mutation and amplification could indicate oncogene addiction, but since targeted therapy for *KIT*-mutated AM and MM is generally not licensed and not available for broad use, it is challenging to obtain samples to validate our study. However, these considerations could be taken into account for future clinical trials design.

The variety of *KIT* alterations including complex mutations across multiple exons with or without gene amplification creates a complicated scenario for successful targeting of KIT protein in melanoma.^50^^,^^51^ Also, similar to previous observations with imatinib,^40^ the same mutations were associated with variable responses in different patients, which may suggest a complex interaction between multiple oncogenic pathways. In contrast, *KIT* alterations in gastrointestinal stromal tumors are more homogeneous, with 70%–90% being exon 11 deletions, and potentially relatedly, KIT inhibitors are an effective standard of care across most patients with *KIT*-mutated gastrointestinal stromal tumors. *KIT* aberrations in AM and MM include hotspot point mutations at the juxta membrane and tyrosine kinase domain, respectively (L576P (Ex 11) and K642E (Ex 13)) as well as complex in or out of frame indels or duplications involving exons 11, 13, and 17 (kinase domain).

Our approach facilitated the detection of these complex variants, which would not be discovered by hotspot assays. Consistent with literature reports, most mutations were localized in exon 11 (n = 20, 69%), and the most common mutation was L576, observed in nine patients (31%); and we showed that tumor responses are not restricted to exon 11 mutations. Our findings have relevant ramifications for *KIT* testing strategies, because despite the availability of tests with broader capture of *KIT* alterations, most *KIT* tests still in use in clinic for economic reasons fail to detect non-L576 or non-exon 11 mutations, thus missing patients who could benefit from KIT-targeted treatment. We suggest that an extended assessment of *KIT* to detect indels and complex aberrations across exons 11, 13, and 17 would provide a useful therapeutic option for patients who have no therapeutic alternatives and whose tumors harbor *KIT* mutations currently undetected. This could pose concerns about the high cost of genetic sequencing,^52^ and the availability of tissue could be an additional limit. This is particularly important given the high number of patients that would need to be screened for *KIT* variants, and also the possible limited quality outputs when using archival FFPE samples to test *KIT* amplifications with alternative methods such as gene sequencing or FISH. However, these limitations should be considered in the context of the scarce alternative therapeutic options and limited benefit from ICB that these patients have. Based on our results, we recommend the use of technologies that, albeit more expensive, enable a more complete detection of *KIT* alterations in a clinical setting.

### Limitations of the study

Based on our results, suggesting a prognostic value of plasma mk-CN, we hypothesize that concomitant mutation and amplification could indicate oncogene addiction. However, we could not verify this hypothesis *in vitro* and could not obtain additional patient samples to validate our study because targeted therapy for KIT-mutated AM and MM is generally not licensed and not available for broad use.

### Conclusion

Nilotinib has an activity comparable to what has been reported for other *KIT* inhibitors and is a viable therapeutic option, including for *KIT* mutations not captured in current standard protocols. ddPCR-based *KIT* analysis appears feasible and accurate for *KIT* testing in patients with metastatic MM and AM and could be proposed for liquid biopsies testing.

## STAR★Methods

### Key resources table

**Table:** 

REAGENT or RESOURCE	SOURCE	IDENTIFIER
**Biological samples**		

FFPE tumor samples	Patients	N/A
Plasma samples	Patients	N/A

**Critical commercial assays**		

Droplet digital Polymerase Chain Reaction SuperMix for probes	BioRad	Cat #1863024
Droplet digital Polymerase Chain Reaction primers and FAM/HEX probes	BioRad	cat #10031276, #10031279, #10049550, #10049047
Fluorescent probes for chromosome 4 centromer (5-fluoreshein (FITC), and *KIT* (5-tamra)	Pishes Empire	Cat # KIT-CHR04-20- ORGR
QIAamp Circulating Nucleic Acid Kits	Qiagen	Cat #55114
Agilent SureSelect sample preparation protocol V2	Agilent	https://www.agilent.com/cs/library/brochures/SureSelect%20CREV2%20Brochure%205991-7572EN%204.9%20(Single%20Page).pdf
Agilent SureSelect sample preparation protocol V4	Agilent	https://www.agilent.com/cs/library/flyers/Public/5990-9857en_lo.pdf

**Software and algorithms**		

STATA v13 & later	StataCorp	https://www.stata.com/
R package OneArmPhaseTwoStudy (run in R version 4.1.3)	Kieser et al.^53^	N/A
BWA	Li et al.^54^	https://github.com/lh3/bwa
Samtools	Li et al.^55^	http://www.htslib.org
Picard	http://picard.sourceforge.net/index.shtml	
SomaticSniper	Larson et al.^56^	https://gmt.genome.wustl.edu/packages/somatic-sniper/documentation.html
Strelka	Saunders et al.^53^	https://github.com/Illumina/strelka
CREST	Wang J et al.^57^	
GATK	McKenna et al.^58^	https://gatk.broadinstitute.org/hc
Varscan	Koboldt et al.^59^	https://varscan.sourceforge.net
SomaticIndelDetector	McKenna et al.^58^	http://www.broadinstitute.org/gatk/gatkdocs/org_broadinstitute_sting_gatk_walkers_indels_SomaticIndelDetector.html
Ensembl Variant Effect Predictor	McLaren et al.^60^	https://www.ensembl.org/vep

### Resource availability

#### Lead contact

Further information and requests for resources should be directed to the lead contact, Prof Samra Turajlic, Skin and Renal Units, The Royal Marsden Hospital NHS Foundation Trust, London, UK (samra.turajlic@crick.ac.uk).

#### Materials availability

There is no availability of biological material because we utilised unique patient samples that were utilized in their entirety. This study did not generate new unique reagents and the ddPCR primer sequences are available from BioRad Assay Design Tool by inputting the *KIT* alteration sequences.

#### Data and code availability

• The ddPCR primer sequences are available from BioRad Assay Design Tool. De-identified data reported in this paper will be shared upon request; applicants can contact the Lead applicant of the Clinical Trials and Statistics Unit at the Institute of Cancer Research (ICR-CTU), who coordinated this study. Trial data are collected, managed, stored, shared, and archived according to ICR-CTSU Standard Operating Procedures to ensure the enduring quality, integrity, and utility of the data. Formal requests for data sharing are considered in line with ICR-CTSU procedures with due regard given to funder and sponsor guidelines. Requests are via a standard proforma describing the nature of the proposed research and extent of data requirements. Data recipients are required to enter a formal data sharing agreement that describes the conditions for release and requirements for data transfer, storage, archiving, publication, and intellectual property. Restrictions relating to patient confidentiality and consent will be limited by aggregating and anonymising identifiable patient data. Additionally, all indirect identifiers that could lead to deductive disclosures will be removed in line with Cancer Research UK Data Sharing Guidelines. Further information can be found here: https://www.icr.ac.uk/our-research/centres-and-collaborations/centres-at-the-icr/clinical-trials-and-statistics-unit/working-with-us/data-sharing.
• This paper does not report original code.
• Any additional information required to reanalyze the data reported in this work paper is available from the lead contact upon request.

### Experimental model and study participant details

NICAM is a multicentre, open-label, investigator-initiated, single-arm two-stage phase 2 study conducted across 16 UK sites (Table S6). Eligible patients were 18 years or older, with *KIT* mutated histologically proven advanced (unresectable locally advanced or metastatic) mucosal or acral melanoma. Patients whose tumors harbored *KIT* mutation previously characterised as conferring resistance to nilotinib were excluded. Patients were required to have one or more clinically or radiologically measurable lesions (≥10mm), Eastern Cooperative Oncology Group (ECOG) performance status 0–2, and adequate organ function. Patients with intracranial disease were excluded (unless present and stable for >6 months). Prior exposure to tyrosine kinase inhibitors was excluded. The full list of inclusion and exclusion criteria are provided in supplementary Table S6.

Patients provided written informed consent before enrollment; initially for *KIT* mutation screening and, once eligibility was confirmed, for entry into the treatment stage of the trial.

### Method details

#### Pre-screening

*KIT* mutation status was ascertained from the genomic DNA extracted from formalin fixed paraffin embedded tumor tissue (either archived or obtained for the purpose of trial screening). Exons 9, 11, 13 and 17 were evaluated by PCR amplification, followed by Capillary Electrophoresis Single-Strand Conformation Analysis (CE-SSCA) and direct Sanger sequencing for identification of the exact mutation. CE-SSCA for *KIT* detects >95% of mutations with a limit of detection of 5–10%, while direct sequencing has a limit of detection of 20–30%. Most analyses were conducted by a central accredited laboratory at The Royal Marsden NHS Foundation Trust. Sites with a laboratory accredited to perform *KIT* mutational analysis also performed *KIT* gene sequencing and analyses, but all reports were centrally reviewed. The suitability of the patient to enter the study based on the mutational profile were determined by the chief investigator. Patients whose tumors were found to harbor *KIT* mutation were eligible for the trial. Patients whose tumors were wild type for *KIT* or did not enter the trial for any reason were treated according to local protocols.

#### Trial procedures

All patients who were included in the NICAM study received oral nilotinib (two 200 mg capsules) twice a day (800 mg per day in total) in 4-week cycles for as long as there was evidence of clinical benefit; treatment beyond radiological progression was allowed. Patients attended for visits on days 1, 15, 29, 57 and then every 4 weeks in year 1; and 8 weekly thereafter for as long as they were receiving trial treatment and were able to attend. Patients underwent CT scans of the thorax, abdomen and pelvis for tumor assessment at screening and after 12 and 26 weeks following initiation of treatment. Further CT scans were performed 3-monthly until 3 years, and 4-monthly thereafter, until progression of disease. Adverse events were recorded according to the National Cancer Institute Common Terminology Criteria (NCI-CTC) version 3. Guidance on drug interruptions or dose reductions for relevant haematological and non-haematological toxicities were implemented as outlined in the protocol. After treatment discontinuation, patients were followed for survival status.

#### Translational analyses

Whole EDTA blood samples were collected pre-treatment (baseline), 2 weeks after start of nilotinib and at disease progression. Formalin fixed paraffin embedded (FFPE) tumor blocks were also available for exploratory analyses where patients provided additional consent.

#### Genomic DNA isolation

Genomic DNA was isolated as described previously^47^^,^^61^; in brief, DNA was extracted from plasma using QIAamp Circulating Nucleic Acid Kits (Qiagen) and quantified with Qubit Assay (Thermofisher Scientific). Based on the *KIT* mutation determined during screening custom primers and probe sets were designed using BioRad Assay Design Tool; BioRad ddPCR assays utilised ddPCR Supermix for probes (cat 1863024) and FAM/HEX kits (cat 10031276, 10031279, 10049550, 10049047). Wild type and mutant alleles in the tumor and circulating tumor DNA (ctDNA) were quantified by ddPCR; custom drop-off probes were designed to detect complex mutations that would not be detected by standard ddPCR assays.^62^ The specificity of the primer/probes was tested using healthy donor peripheral blood mononuclear cells’ DNA as negative control, and patient-matched tumor DNA was used as positive control.

#### Allele quantification

The amount of mutant and wild type DNA in each sample was quantified using Bio-Rad QX200 platform and expressed as variant allele frequency (VAF, the fraction of mutant droplets in the total number of mutant and wild-type droplets). Mutated *KIT* copy number (mK-CN, the fraction of mutant *KIT* droplets over the number of droplets positive for the reference gene *hTERT*), was calculated utilising the median values of three technical replicates as previously described.^47^

#### FISH

*KIT* gene amplification confirmation was exploratorily tested in FFPE archival tumor samples by means of FISH, that was performed with dapi staining for nuclei and Pishes Empire fluorescent probes for chromosome 4 centromer (5-fluoreshein (FITC), and *KIT* (5-tamra) using the producer’s protocols; the stained slides were evaluated on a Zeiss Imager.M1, AX10 or Zeiss M200 FL microscope.

#### Whole genome/exome sequencing (WGS/WES)

Exploratory WGS/WES was pursued in a small subset of NICAM patients co-enrolled in tissue biobanking study.^3^ For WGS, DNA was sequenced using Illumina Hiseq2000 sequencers, the FASTQ files of the paired-end reads were aligned to the human reference genome (GRCh37) and processed using default settings BWA,^54^ Samtools^55^ and Picard (https://broadinstitute.github.io/picard/). We used SomaticSniper (score threshold ≥40, a mapping threshold ≥40, and depth in tumor and normal ≥10) to call the somatic single nucleotide variants (SNVs),^56^ applying pre-determined filters to remove likely false-positive SNVs 20.^59^ Somatic indels were called using Strelka^53^ removing low-confidence indels. All SNVs and indels were annotated,^60^ and SNVs and indels present in dbSNP 135 were excluded. We used Illumina’s cancer pipeline to identify copy number alterations (CNAs) and assessed the somatic structural variations with CREST^57^ (default settings for comparison between normal and tumor).

Whole human exome capture and sequencing was performed using Agilent SureSelect sample preparation protocol V2 (37 Mb) with Illumina GAIIX sequencer (76 bp paired-end reads) or Agilent SureSelect sample preparation protocol V4 (50 Mb) with HiSeq 2000 sequencer (100 bp paired-end reads). Sequences were aligned to the NCBI build 37 reference genome using BWA^54^ and processed with Picard and GATK.^58^ Somatic SNVs were called using Varscan with predetermined filters to remove false positives^59^ and SomaticSniper.^56^ We used SomaticIndelDetector to identify somatic indels (https://gatk.broadinstitute.org/hc/en-us) and Ensembl Variant Effect Predictor to annotate somatic variants.^60^

#### Outcomes

The primary endpoint was the proportion of patients who were alive and progression free at six months according to RECIST 1.1.^63^ Progression free survival (PFS) was measured from the date of enrollment into the treatment phase until the first date (following start of treatment) of either death or confirmed progressive disease according to RECIST 1.1. The secondary endpoints of the study included OR rate (complete or partial response as per RECIST 1.1) at 12 weeks, OS (measured from the date of enrollment until the date of death due to any cause) and the safety and tolerability profile of nilotinib. Post-hoc exploratory endpoints included assessment of the primary endpoint as reviewed centrally, and proportion of patients presenting indolent disease at trial entry as ascertained by central assessment of pre-baseline (within three months of trial entry) and baseline scans. The presence of indolent disease can impact interpretation of drug effectivenessparticularly in this non-randomised trial. Indolent disease was defined as stable disease or lesion growth <20% between pre-baseline and baseline scans. Translational secondary endpoints were the association of particular *KIT* mutations and *KIT* gene amplification with response to treatment and survival.

### Quantification and statistical analyses

Efficacy endpoints were reported in the subgroup of patients considered evaluable for the primary endpoint assessment. Safety was reported on all patients who received at least one dose of study drug.

A cohort of 24 evaluable patients was targeted under a two-stage design (nine in stage one, 15 in stage two), where there would be an 86% power for nilotinib to show sufficient activity (≥15%) to pursue further investigation (one-sided alpha = 5%) if the true proportion of patients progression-free at six months was 40%. At least 2/9 and 7/24 patients to be progression-free at 6 months were required as success criteria at stage one and two, respectively. To account for the two-stage design, the 2-sided 90% confidence interval for PFS at six months and p value for decision making were obtained as per Koyama and Chen (2008).^64^ The PFS at six months was also estimated by the uniformly minimum variance unbiased estimator (UMVUE) to account for the two-stage design.^65^ The R library OneArmPhaseTwoStudy was used to obtain these adjusted parameters (R version 4.1.3).^66^ Given that the trial over-recruited to account for non-evaluable patients, these estimates were also obtained for the whole evaluable cohort.

Kaplan-Meier estimates for PFS and OS were graphically summarised in survival curves. Response rates were summarised with 95% exact binomial confidence intervals. Most common (by NCI-CTC grade), dose-limiting and serious adverse events and reactions were summarised by frequencies and percentages. As exploratory analysis, we analyzed the association between disease burden at baseline (as measured by sum of target lesions) and PFS and OS with Cox Proportional Hazards models.

Association of mutations and amplification with OR and best change from baseline in tumor size at 12 weeks were summarised descriptively, and groups compared by appropriate non-parametric tests (i.e., Kruskal-Wallis or Mann-Whitney, respectively). Cox proportional hazard models were used to quantify association of continuous biomarkers with PFS and OS. Exploratory cut-offs based on the median of the biomarkers were used to categorise them, as no clear clusters of data were observed. Kaplan-Meier estimates of the survival function for each biomarker category (amplified vs. non-amplified as per the median value) were graphically presented and compared by log rank tests. Correlations between tumor and plasma DNA, and with baseline disease burden were measured by Spearman correlation coefficient. Due to the small number of patients, the p values presented are considered hypothesis-generating.

Statistical analyses were done with Stata software (version 13 & later), on a snapshot of the clinical data taken on 9 January 2017, when all patients have completed trial follow-up. Biological and biomarker data for translational analyses presented in this report were generated after trial completion.

### Additional resources

The study was approved by the Oxfordshire Research Ethics Committee (REC ref. 09/H0606/103), and co-sponsored by The Royal Marsden NHS Foundation Trust and The Institute of Cancer Research (ICR), London, UK. The trial was conducted in accordance with the principles of good clinical practice and overseen by an Independent Data Monitoring and Steering Committee. A Trial Management Group (TMG) was responsible for the day-to-day running of the trial. The Clinical Trials and Statistics Unit at ICR (ICR-CTSU) had overall responsibility for trial coordination, monitoring, and data analysis.

Trial registration: ISRCTN39058880, EudraCT 2009-012945-49.

## Supplemental information

Document S1. Figures S1–S6 and Tables S1–S6

Document S2. Article plus supplemental information
