# Supplementary material for: Nilotinib in KIT-driven advanced melanoma: Results from the phase II single-arm NICAM trial
Source: Cell Rep Med. 2024 Feb 27;5(3):101435. doi: 10.1016/j.xcrm.2024.101435 (PMC10982988; doi:10.1016/j.xcrm.2024.101435)
Supplement: Document S1. Figures S1–S6 and Tables S1–S6 [file mmc1.pdf]

Cell Reports Medicine, Volume *5*

Supplemental information

Nilotinib in *KIT*-driven advanced melanoma:

Results from the phase II single-arm NICAM trial

James Larkin, Richard Marais, Nuria Porta, David Gonzalez de Castro, Lisa Parsons, Christina Messiou, Gordon Stamp, Lisa Thompson, Kim Edmonds, Sarah Sarker, Jane Banerji, Paul Lorigan, Thomas R. Jeffry Evans, Pippa Corrie, Ernest Marshall, Mark R. Middleton, Paul Nathan, Steve Nicholson, Christian Ottensmeier, Ruth Plummer, Judith Bliss, Sara Valpione, and Samra Turajlic

Supplementary Tables

Table S1: Baseline characteristics of all patients screened in NICAM
Related to Figure 1 and Table 1

	**Patients screened (N=218[a])**	
	**N**	**%**
**Patient demographics**		
Sex		
Female	135	61.9
Male	80	36.7
Unknown	3	1.4
Age at registration/entry (yr), mean(SD)	65.6 (12.1)	
Ethnicity		
Caucasian	185	84.9
Asian	6	2.8
Other	10	4.6
Unknown	17	7.8
Skin type (Fitzpatrick classification)		
I	14	6.4
II	21	9.6
III	96	44
IV	10	4.6
V	4	1.8
VI	5	2.3
Unknown	68	31.2
**Melanoma subtype**		
Acral	67	30.7
Location		
Finger	*5*	*2.3*
Heel	*8*	*3.7*
Instep	*1*	*0.5*
Sole (non specific type)	*21*	*9.6*
Subungual (Foot)	*4*	*1.8*
Subungual (Hand)	*3*	*1.4*
Toe	*24*	*11*
Stage at presentation		
Localised	*44*	*20.2*
Regional lymph node metastasis	*7*	*3.2*
Distant metastasis	*12*	*5.5*
Unknown	*4*	*1.8*
Mucosal	151	69.3
Location		
Head and neck	*48*	*22*
Upper gastrointestinal tract	*10*	*4.6*
Anorectal	*30*	*13.8*
Urogenital	*56*	*25.7*
Upper respiratory tract	*2*	*0.9*
Other[b]	*6*	*2.8*
Stage at presentation		
Localised I	*41*	*18.8*
Localised II	*42*	*19.3*
Localised III	*22*	*10.1*
Unknown	*46*	*21.1*

[a]No baseline features available for one patient screened for c-kit mutation (but not entered) [b]Includes lower gastrointestinal tract (n=3 lower respiratory tract (n=1), eyes (n=1) and unknown (n=1) Yr: year; SD: standard deviation.

Table S2: List of NICAM patients - *cKIT* mutation details, RECIST response and key endpoints
Related to Table 1, Figures 2,3,4

**ID**	**Melanoma subtype**	**Exon**	***cKIT* mutation**	**mK-CN (biopsy)**	**RECIST - Baseline sum of target lesions (cm)**	**RECIST – best % change in sum of target lesions within 12 weeks**	**Months on treatment**	**Months to progression**	**Months to death**
NI01	Mucosal	11	c.1658A>C; p.Tyr553Ser	1.2	7.3	12.3	2.9	2.7	6.4
NI02	Mucosal	11	c.1668_1739del72; p.Gln556_Asp579del	3.4	2.8	-28.6	2.7	2.7	32.4
NI03	Mucosal	11	c.1676T>A; p.Val559Asp	10.6	12	-14.2	16.5	5.8	27.9
NI04	Mucosal	11	c.1679T>A , p.Val560Asp	7.2	2.4		0.6	0.9	2.2
**NI05**	**Mucosal**	**11**	**c.1716_1733dup; p.573_578dup**		**6.5**	**0**	**30.7**	**15.1**	**32.7**
NI06	Acral	11	c.1716_1736del; p.Pro573_Asp579del	3.3	2.1*		17.4	2.7	20.8
**NI07**	**Mucosal**	**11**	**c.1727T>C; p.Leu576Pro**	**5.4**	**5**	**2**	**34.5**	**32.9**	**39.9**
**NI08**	**Mucosal**	**11**	**c.1727T>C; p.Leu576Pro**	**13**	**2.1**	**-90.5**	**17.0**	**16.8**	**17.3**
**NI09**	**Mucosal**	**11**	**c.1727T>C; p.Leu576Pro**	**4.4**	**20.2**	**3.5**	**10.6**	**8.5**	**15.8**
NI10	Mucosal	11	c.1727T>C; p.Leu576Pro	12.3	15.8		1.4	1.2	1.5
NI11	Mucosal	11	c.1727T>C; p.Leu576Pro	2.9	6.2	-1.6	10.2	5.9	10.3
NI12	Mucosal	11	c.1727T>C; p.Leu576Pro	5.9	21.3	30	3.2	2.9	5.3
NI13	Acral	11	c.1727T>C; p.Leu576Pro	0.8	3.2	109.4	2.8	2.8	2.8+
NI14	Mucosal	11	c.1727T>C; p.Leu576Pro		9	-44.4	2.2	2.6	3
NI15	Acral	11	c.1730_1732del; p.Pro577_Tyr578delinsHis	3.6	12.5	1.6[¥]	1.6	1.7	2.8
NI16	Mucosal	11	c.1732_1773dup; p.Tyr578_Phe591dup	1.2	19.9	-15.1	7.4	5.3	7.7
**NI17**	**Mucosal**	**11**	**c.1733A>C; p.Tyr578Ser**	**3.5**	**4.3**	**2.3**	**11.7**	**11.6**	**13.8**
NI18	Mucosal	11	c.1735_1737del, p.Asp579del	10.1	1.4	-71.4	50.6	5.9	61.4
NI19	Mucosal	11	c.1739_1774dup; p.His580_Gly592complex	13.2	9.7	40.2[¥]	1.8	1.6	2.7
NI20	Acral	13	c.1924A>G; p.Lys642Glu		3.9	-7.7	6.5	6.3	12.6
NI21	Acral	13	c.1924A>G; p.Lys642Glu		4.6	39.1	2.4	2.3	4.1
NI22	Mucosal	13	c.1924A>G; p.Lys642Glu	1.4	4.9	-38.8	5.7	6.1	6.1
NI23	Acral	13	c.1965T>G; p.Asn655Lys	1.3	2.6	69.2	1.0	2.1	5.1
**NI24**	**Mucosal**	**17**	**c.2459A>T; p.Asp820Val**	**0.7**	**6.3**	**-38.1**	**54.2**	**15.6**	**63.7**
NI25	Mucosal	17	c.2460T>A; p.Asp820Glu	5.6	9.1	-2.2	3.7	3.7	7.1
NI26	Mucosal	17	c.2464A>T; p.Asn822Tyr	1	11.9	-68.9	5.5	5.4	6.5
NI27	*Mucosal*	*11*	*c.1727T>C; p.Leu576Pro*	*1.8*	*4.2*		*2.2*	*4.4*	*19.1*
NI28	*Mucosal*	*9*	*c.1504_1509dup; p.Ala502_Tyr503dup*	*13.6*	*22.3**		*0.9*	*1.4§*	*1.4§*
NI29	*Mucosal*	*17*	*c.2466T>A; p.Asn822Lys*				*0*	*0*	*0.5*

mK-CN: mutated *KIT* copy number (measured on baseline biopsy sample). RECIST: Response Criteria for Solid Tumours 1.1 as per central review, except for * where this was not available, and local assessment is reported instead. In bold, patients alive and progression free as per local assessment (primary endpoint). Patient NI11 was considered alive and progression free as per central review. Patients NI27, NI28, NI29 were not evaluable for the primary endpoint. [¥] Reported at progression<12 weeks. +Patient alive at last follow-up (lost to follow-up after progression). §Patient alive and progression free at last follow-up (withdrew from trial assessments). Cases with the same c-KIT mutation are highlighted in grey.

Table S3: *cKIT* mutation detail, by type of mutation and mutated *KIT* copy number amplification
Related to Figures 2,3,4

		**mk-CN<p50 (non-amplified)**		**mK-CN ≥p50 (amplified)**		**Total**	
	**Exon**	**n**	**%**	**n**	**%**	**n**	**%**
Complex insertion or deletion		3	27.3	3	27.3	6	27.3
c.1668_1739del72; p.Gln556_Asp579del	*11*	*1*	*9.1*	*0*	*0*	*1*	*4.5*
c.1716_1736del; p.Pro573_Asp579del	*11*	*1*	*9.1*	*0*	*0*	*1*	*4.5*
c.1730_1732del; p.Pro577_Tyr578delinsHis	*11*	*0*	*0*	*1*	*9.1*	*1*	*4.5*
c.1732_1773dup; p.Tyr578_Phe591dup	*11*	*1*	*9.1*	*0*	*0*	*1*	*4.5*
c.1735_1737del, p.Asp579del	*11*	*0*	*0*	*1*	*9.1*	*1*	*4.5*
c.1739_1774dup; p.His580_Gly592complex	*11*	*0*	*0*	*1*	*9.1*	*1*	*4.5*
Missense mutation		8	72.7	8	72.7	16	72.7
c.1658A>C; p.Tyr553Ser	*11*	*1*	*9.1*	*0*	*0*	*1*	*4.5*
c.1676T>A; p.Val559Asp	*11*	*0*	*0*	*1*	*9.1*	*1*	*4.5*
c.1679T>A ; p.Val560Asp	*11*	*0*	*0*	*1*	*9.1*	*1*	*4.5*
c.1727T>C; p.Leu576Pro	*11*	*2*	*18.2*	*5*	*45.5*	*7*	*31.8*
c.1733A>C; p.Tyr578Ser	*11*	*1*	*9.1*	*0*	*0*	*1*	*4.5*
c.1924A>G; p.Lys642Glu	*13*	*1*	*9.1*	*0*	*0*	*1*	*4.5*
c.1965T>G; p.Asn655Lys	*13*	*1*	*9.1*	*0*	*0*	*1*	*4.5*
c.2459A>T; p.Asp820Val	*17*	*1*	*9.1*	*0*	*0*	*1*	*4.5*
c.2460T>A; p.Asp820Glu	*17*	*0*	*0*	*1*	*9.1*	*1*	*4.5*
c.2464A>T; p.Asn822Tyr	*17*	*1*	*9.1*	*0*	*0*	*1*	*4.5*
**Total**		**11**	**100**	**11**	**100**	**22**	**100**

mK-CN: mutated *KIT* copy number; highlighted cells represents occurrences with patients with PFS≥6 months (3/5 patients for *c.1727T>C; p.Leu576Pro* and mK-CN amplified):

PFS>=6m
3/5 PFS>=6m

Table S4. Summary of the molecular analyses performed on the tumour biopsy samples and clinical outcomes

Related to Figure 2

ID[1]	*cKIT* mutation	mK-CN (biopsy)	WGS ID[2]	WES ID[2]	FISH[3]	RECIST – % change in sum of target lesions within 12 weeks	Months on treatment	Months to progression
NI02	c.1668_1739del72;<br>p.Gln556_Asp579del	3.4		N10213		-28.6	2.7	2.7
NI03	c.1676T>A; p.Val559Asp	10.6	N05408		CEN4/nucleous~2-4<br>*KIT*:CEN4>1<br>*KIT*:nucleus~2-4	-14.2	16.5	5.8
NI10	c.1727T>C; p.Leu576Pro	12.3			CEN4:nucleous~3<br>*KIT*:CEN4=1<br>*KIT*:nucleus>2		1.4	1.2
NI11	c.1727T>C; p.Leu576Pro	2.9			Heterogeneous<br>CEN4:nucleous~3<br>*KIT*:CEN4=1<br>*KIT*:nucleus>2	-1.6	10.2	5.9
NI12	c.1727T>C; p.Leu576Pro	5.9			Heterogeneous; some areas are euploids and *KIT*:CEN4=1, others appear<br>CEN4:nucleous~2-5<br>*KIT*:CEN4~1<br>*KIT*:nucleus~2-5	30	3.2	2.9
NI15	c.1730_1732del;<br>p.Pro577_Tyr578delinsHis	3.6			Very heterogeneous, some areas are euploidy with *KIT*:CEN4=1, others<br>CEN4:nucleous=1<br>*KIT*:CEN4=~4<br>*KIT*:nucleus~4	1.6[¥]	1.6	1.7
NI16	c.1732_1773dup;<br>p.Tyr578_Phe591dup	1.2	N01803			-15.1	7.4	5.3
NI18	c.1735_1737del, p.Asp579del	10.1		N06610		-71.4	50.6	5.9
NI19	c.1739_1774dup;<br>p.His580_Gly592complex	13.2		N01502		40.2[¥]	1.8	1.6
NI22	c.1924A>G; p.Lys642Glu	1.4			Euploid and *KIT*:CEN4=1	-38.8	5.7	6.1
NI27	c.1727T>C; p.Leu576Pro	1.8		N00101			2.2	4.4

[1]All Mucosal type except NI15 acral; all Exon 11 except NI22 (exon 13; NI27 considered not evaluable for primary endpoint analysis

[2]Whole genome sequencing (WGS) and whole exome sequencing (WES) identifiers (ID) used in a in a small subset of NICAM patients co-enrolled in a tissue biobanking study (Furney, Turajlic et al., Journal of Clinical Pathology, 2013) . Results are not reproduced here to avoid data duplication

[3]The count of probe signals for KIT and the centromere of chromosome 4 (CEN4) per nucleous in cancer cells for the 6 samples that could be analysed with FISH. Some cells appear to have duplications of both KIT and centromere of chromosome 4, others to have duplications of KIT with normal number of centromere of chromosome 4.

Table S5. Systematic review of studies of targeted therapies in advanced melanoma harbouring KIT alterations
Related to discussion, Table 1, Figure 4

	Patients (n)	Patients with KIT mutation	RR (%)	OS (median)	PFS (median)	TTP (median)	Length of FU (median)	Interven tion
Kluger 2011	36	36	5	12 .0	2	--	--	Dasatinib
Kim 2008	22	22	5	7.5		1.4		Imatinib
Kalinsky 2017	73	3/51 stage 1<br>22/22 stage 2	5.9 KIT-<br>18.2 KIT+	7.5	2.1		59.5 stage 1<br>23.2 stage 2	Dasatinib
Hodi 2013	24	24	21.0[a]	12.5	3.5	3.7[b]	10.6	Imatinib
Buchbinder 2015	52	13	9.7	7.5[c]	--	2.6[d]	--	Sunitinib
Guo 2011	43	43	23.3	15	3.5	--	12	Imatinib
Carvajal 2011	25[e]	25[e]	16	10.7	--	2.8	--	Imatinib
Carvajal 2015	19	11 Cohort A[f]<br>8 Cohort B[f]	18.2<br>0	14.2<br>4.3	--	3.4<br>2.6	16.2<br>11.7	Nilotinib
Guo 2017	42	42	26.2	18	4.2	--	25.8[f]	Nilotinib
Lee 2015	42	42	16.7	17.5	8.5		12.2	Nilotinib

Length of time reported in months. Abbreviations: OS, overall survival; PFS, progression-free survival; RR, response rate, TTP, time to progression, FU Follow-up; UNK, unknown.

a: RR reported also as 29% but only 21% confirmed response

b: 3.9 months with subset analysis KIT mutations and 3.4 months with amplifications

d: median based on 8.6 KIT-;6.4 KIT+;6.2 KIT UNK

c: median based on 2.8 KIT-;3.2 KIT+;1.8 KIT UNK

e: 28 KIT+ patients overall, only 25 evaluable

f: Cohort A: refractory or intolerant to a prior KIT inhibitor, Cohort B: those with brain metastases

g: reported only for 3 living patients

Table S6. NICAM Inclusion and exclusion criteria (as per Protocol V8)
Related to STAR Methods

**Inclusion Criteria**	
1.	Patients with c-KIT mutated histologically proven advanced mucosal or acral melanoma in which the mutation is not known to be associated with nilotinib resistance.
2.	Advanced mucosal and acral melanoma defined as unresectable locally advanced or metastatic disease
3.	The presence of one or more clinically or radiologically measurable lesions at least 10mm in size
4.	Age 18 or greater
5.	ECOG performance status 0, 1 or 2
6.	Life expectancy greater than 12 weeks
7.	At least 14 days since any major surgery
8.	The capacity to understand the patient information sheet and ability to provide written informed consent
9.	Willingness and ability to comply with scheduled visits, treatment plans, laboratory tests and other study procedures
10.	Women must not be pregnant or lactating with no intention of pregnancy during study treatment. Women of child bearing potential must have a negative serum pregnancy test prior to study entry (even if surgically sterilised). Men and women of childbearing potential must use adequate birth control measures (e.g. abstinence, oral contraceptives, intrauterine device, barrier method with spermicide, implantable or injectable contraceptives or surgical sterilisation) for the duration of the study and should continue such precautions for 6 months after receiving the last study treatment
11.	Serum alanine transaminase (ALT) or serum aspartate aminotransferase ≤2.5 x upper limit of normal (ULN) and total serum bilirubin ≤1.5 x ULN
12.	Serum creatinine ≤1.5 x ULN
13.	Serum lipase and amylase <1.5 x ULN
14.	Haemoglobin ≥9.0 g/dL, absolute neutrophil count ≥1.5 x 109/L, platelets ≥100 x 109/L
15.	Prothrombin time (PT) ≤1.5 x ULN
16.	Able to swallow and retain oral medication.
**Exclusion Criteria**	
1.	Intracranial disease, unless there has been radiological evidence of stable intracranial disease > 6 months. In the case of a solitary brain metastasis, evidence of a disease-free interval of at least 3 months post surgery. All patients previously treated for brain metastases must be stable off corticosteroid therapy for at least 28 days
2.	Women who are pregnant, nursing, or planning to become pregnant during the course of the trial
3.	Men who plan to father a child during the course of the trial
4.	Use of any investigational drug within 30 days prior to screening (both cancer and non cancer treatments)
5.	Use of herbal or chinese medication
6.	Use of therapeutic coumarin derivatives (ie warfarin, acenocoumarol, phenprocoumon)
7.	Significant cardiac disease including patients who have or who are at significant risk of developing prolongation of QTc
8.	Severe and/or uncontrolled medical disease
9.	Known chronic liver disease
10.	Past medical history of chronic pancreatitis
11.	Known HIV infection
12.	Previous radiotherapy to 25% or more of the bone marrow
13.	Radiation therapy in the 4 weeks prior to study entry
14.	Prior exposure to a tyrosine kinase inhibitor
15.	Known lactose intolerance
16.	Any malabsorption syndrome (i.e. partial gastrectomy, small bowel resection, Crohn's disease or ulcerative colitis).

Supplementary Figures

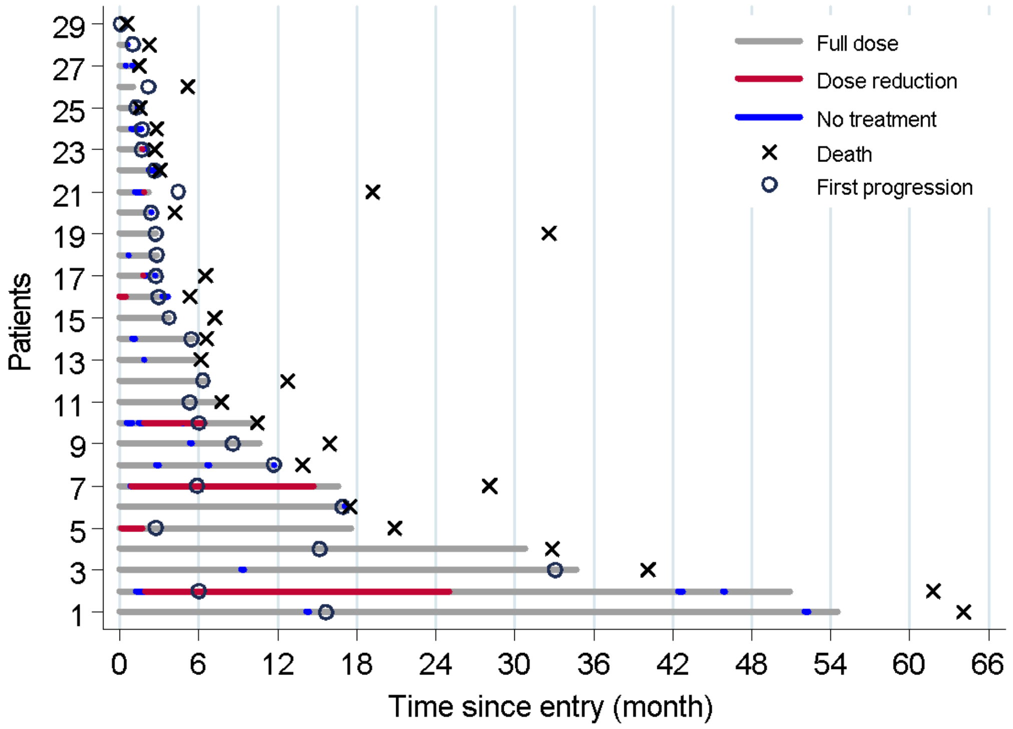

Figure S1: Time on treatment (grey) with periods of dose reduction (red), delay or missing treatment (blue), time of first progression and time of death in all NICAM patients (N = 29)

Bar length indicate months on treatment; objective disease progression and death are indicated in the figure. Patients were allowed to continue treatment as long as clinically indicated by the treating physician.

Related to Figure 3, Table 2

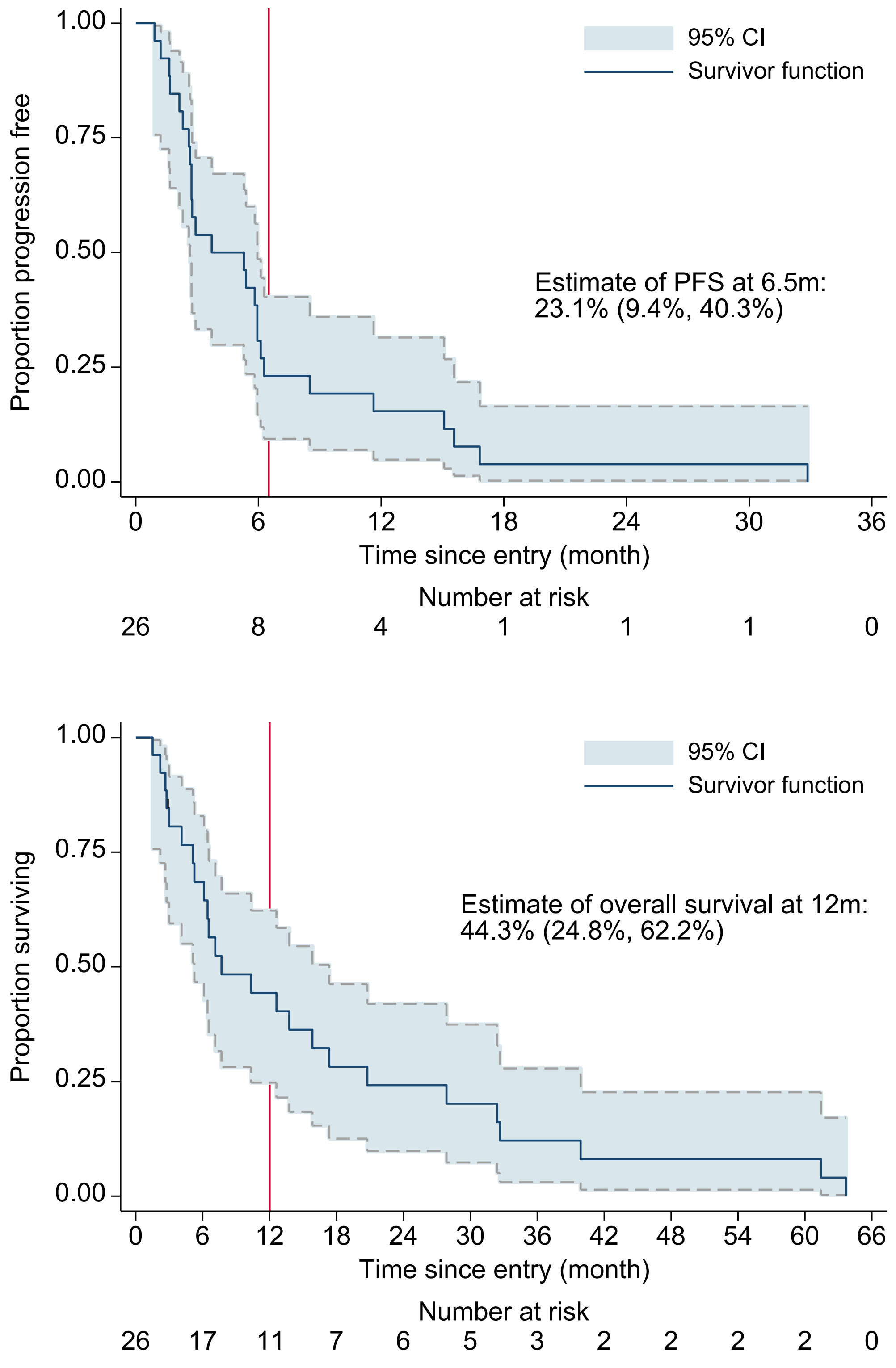

Figure S2: Progression Free Survival (top) and Overall Survival (bottom) Kaplan-Meier estimates on the evaluable population (n=26)

Related to Figures 3, 4

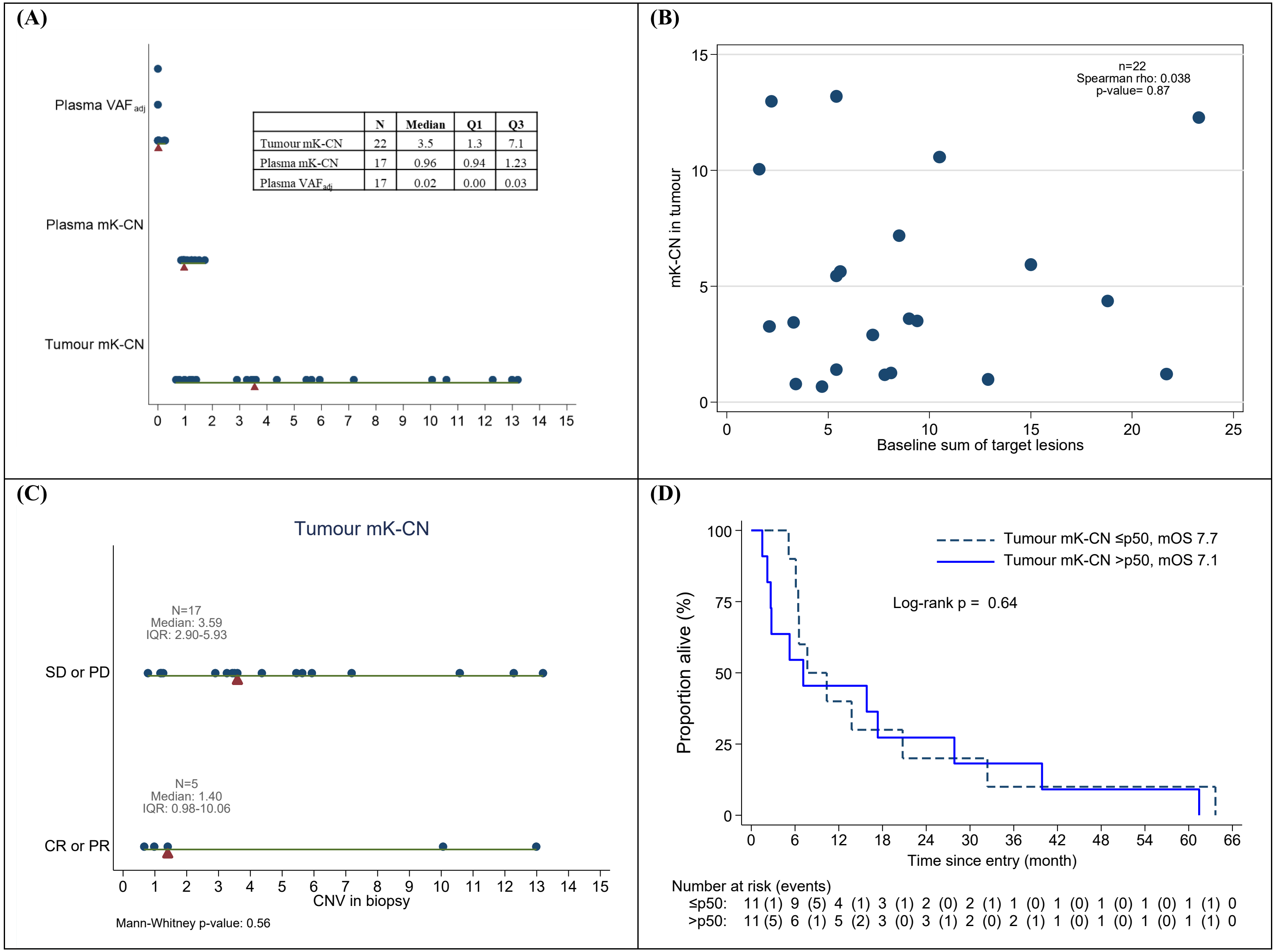

Figure S3 Association of gene amplification in tissue with antitumour activity
(A) Distribution of mK-CN in tumour, mK-CN in plasma and VAF_{adj} in plasma (B) Association of mK-CN in biopsy with disease burden at baseline (represented by sum of target lesions as per RECIST 1.1) (C) Baseline mK-CN in tumour with objective response (RECIST 1.1) at 12 weeks (D) overall survival by baseline mK-CN in tumour, groups defined by its median
mK-CN below median in the analysis set (<p50=3.5); amplified: mK-CN at or above median in the analysis set (≥p50=3.5). All the cfDNA data are the mean of 3 technical replicates for one patient biological sample.
Related to Table 1, Figure 4

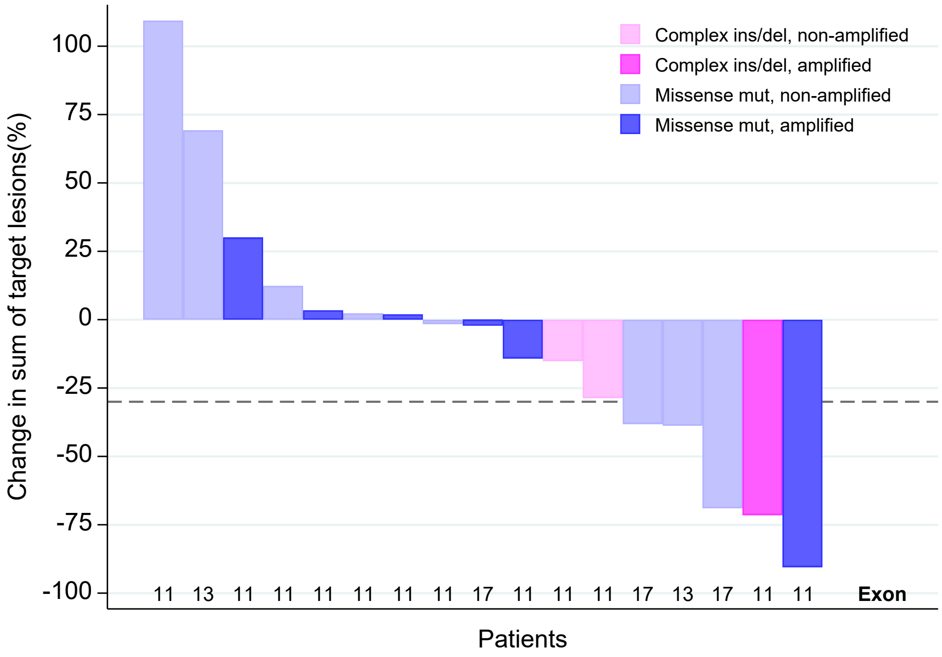

Figure S4. Best percentage change from baseline at 12 weeks in sum of target lesions as per RECIST 1.1 (central review) by type of *KIT* mutation and mutated *KIT* copy number amplification
Complex ins/del= complex insertion or deletion; Missense mut: missense mutation; non-amplified

Related to Figure 4

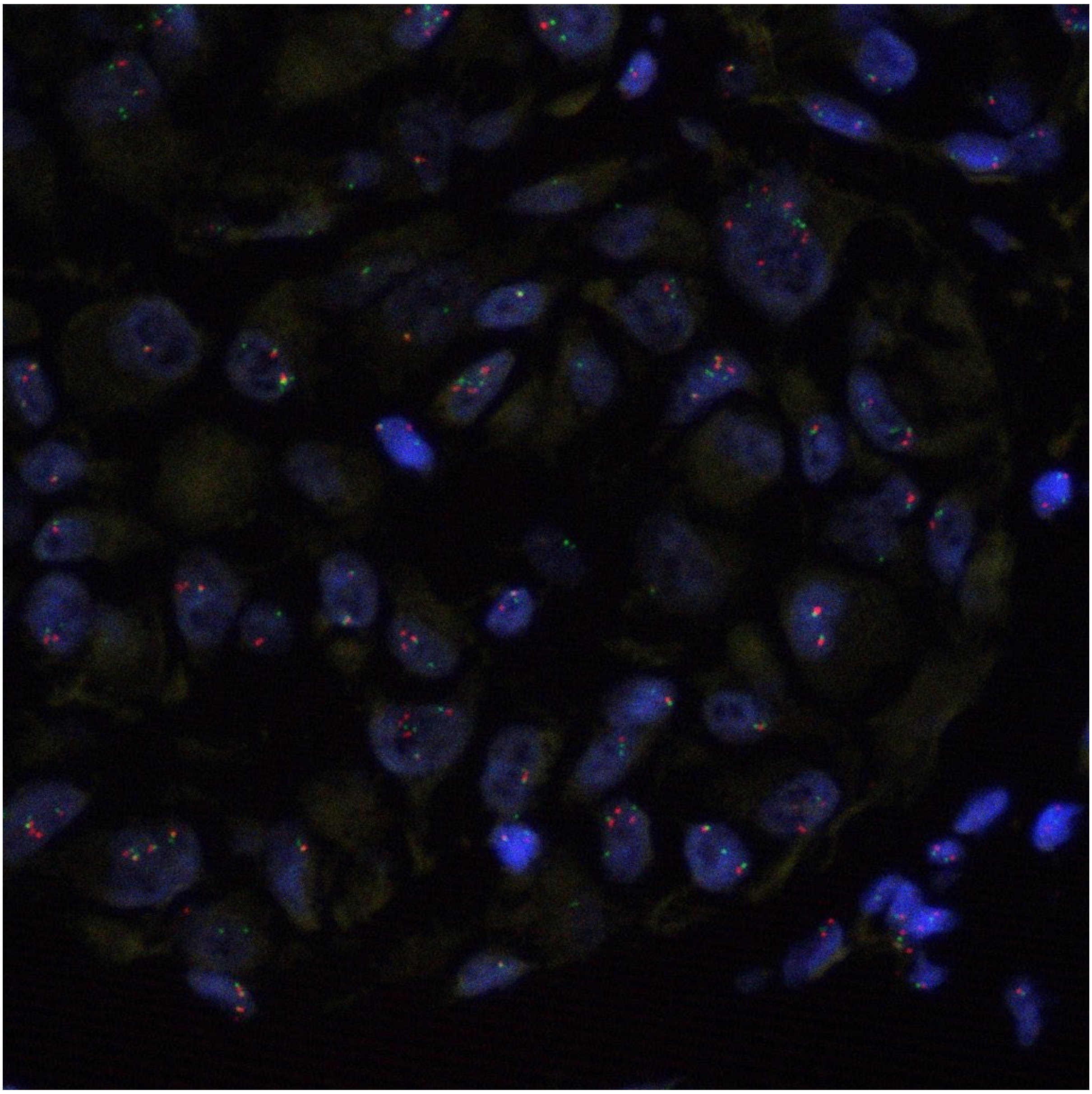

Figure S5. The micrograph shows the fluorescent in situ hybridisation of the paraffin fixed tumour sample of patient NI12 (see Table S4). The nuclei are stained in blue (dapi), the green dots correspond to the chromosome 4 centromere (fluorescein probes) and the orange dots correspond to KIT (temra probes).The white arrows indicate examples of nuclei with two green and two orange dots (diploid for chromosome 4 and KIT), the red arrows highlight examples of cells with more than two copies of chromosome 4 and KIT per nuclei. This patient had mean KIT copies = 5.9 in the tumour and = 1.2 in cfDNA as measured by ddPC

Related to Figure 2, Figure 4

(A)

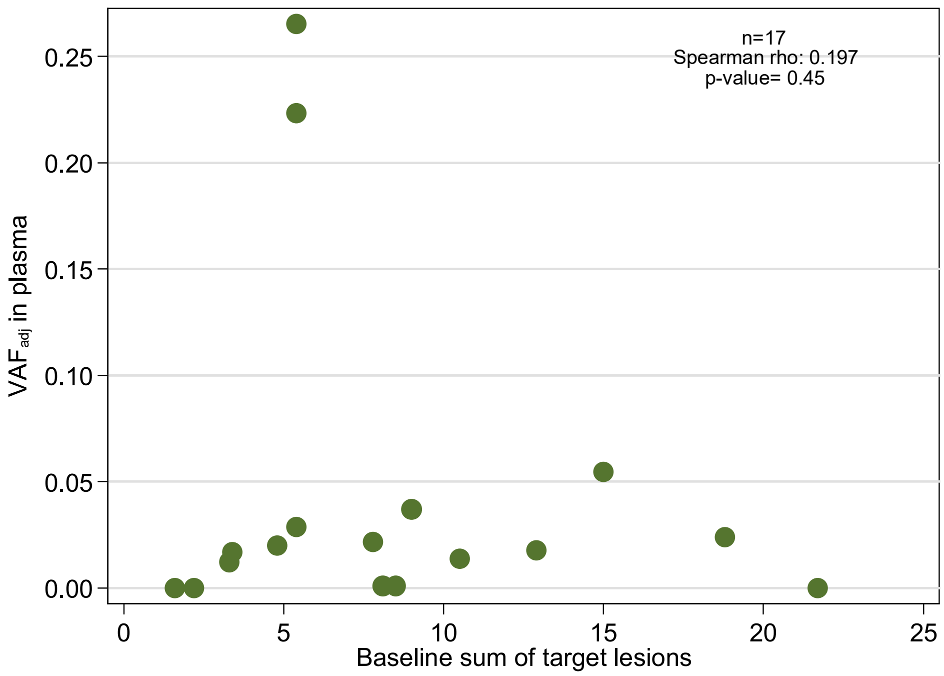

(B)

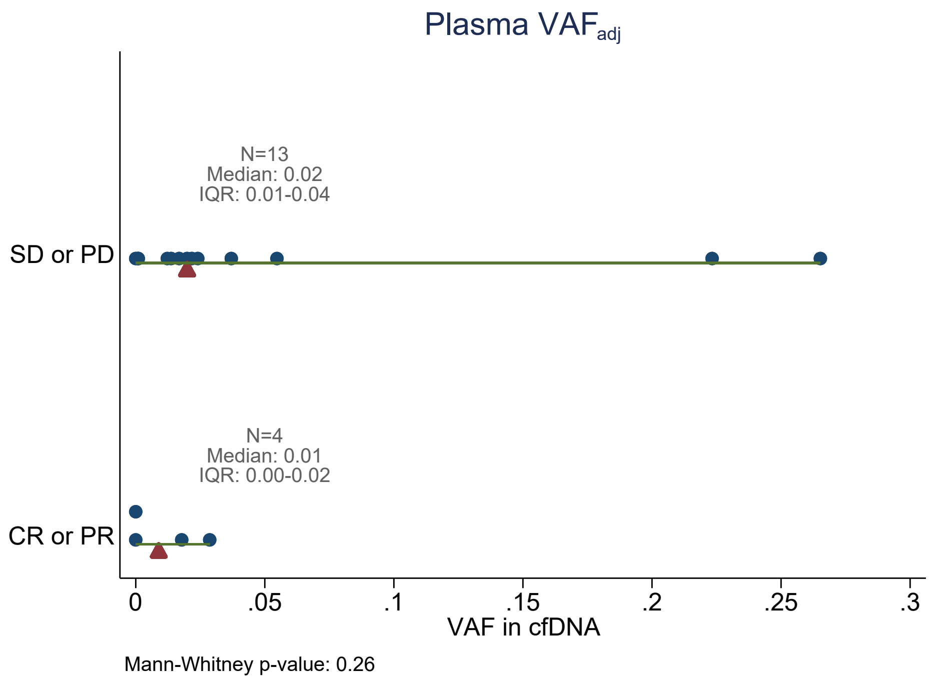

Figure S6: Association of gene amplification in plasma with antitumour activity
(A) Association of VAF_{adj} in blood, with disease burden at baseline (represented by sum of target lesions as per RECIST 1.1) **(B)** Association of Baseline VAF_{adj} in plasma with objective response (RECIST 1.1) at 12 weeks
All the cfDNA data are the mean of 3 technical replicates for one patient biological sample.

Related to Table 1, Figure 4
